# Exploiting aneuploidy-imposed stresses and coping mechanisms to battle cancer

**DOI:** 10.1098/rsob.200148

**Published:** 2020-09-02

**Authors:** Lin Zhou, Laura J. Jilderda, Floris Foijer

**Affiliations:** European Research Institute for the Biology of Ageing, University of Groningen, University Medical Center Groningen, 9713 AV, Groningen, The Netherlands

**Keywords:** chromosomal instability, aneuploidy, cancer, aneuploidy tolerance, intervention

## Abstract

Aneuploidy, an irregular number of chromosomes in cells, is a hallmark feature of cancer. Aneuploidy results from chromosomal instability (CIN) and occurs in almost 90% of all tumours. While many cancers display an ongoing CIN phenotype, cells can also be aneuploid without displaying CIN. CIN drives tumour evolution as ongoing chromosomal missegregation will yield a progeny of cells with variable aneuploid karyotypes. The resulting aneuploidy is initially toxic to cells because it leads to proteotoxic and metabolic stress, cell cycle arrest, cell death, immune cell activation and further genomic instability. In order to overcome these aneuploidy-imposed stresses and adopt a malignant fate, aneuploid cancer cells must develop aneuploidy-tolerating mechanisms to cope with CIN. Aneuploidy-coping mechanisms can thus be considered as promising therapeutic targets. However, before such therapies can make it into the clinic, we first need to better understand the molecular mechanisms that are activated upon aneuploidization and the coping mechanisms that are selected for in aneuploid cancer cells. In this review, we discuss the key biological responses to aneuploidization, some of the recently uncovered aneuploidy-coping mechanisms and some strategies to exploit these in cancer therapy.

## 1. Introduction

Aneuploidy, an abnormal number of chromosomes in cells, affects the majority of cancers, ranging from 26% in thyroid carcinoma to 99% of the glioblastomas and testicular germ cell tumours [1,2]. Aneuploidy is caused by defects in the process of chromosome segregation, collectively referred to as chromosomal instability (CIN) [3]. However, cells can also be aneuploid without exhibiting CIN, for which the most well-known example is Down syndrome, in which cells carry an extra copy of chromosome 21 without a CIN phenotype [4]. The spindle assembly checkpoint (SAC) acts as a safeguard against CIN by delaying anaphase onset until all chromosomes are properly aligned and attached on the metaphase plate [5]. Indeed, defects in *SAC* genes such as *Mad1*, *Mad2*, *BUB3* and *BUBR1* lead to CIN and aneuploidy [6–9]. Also other non-SAC genes have been implicated with CIN phenotypes, including CENP-E [10], SPAG5 [11], Knl1 [12] and many others.

Despite being a hallmark feature of cancer cells, aneuploidy will initially cause growth defects to untransformed cells [13–16]. The fact that aneuploidy is initially toxic to cells but yet frequently occurring in cancer is referred to as the aneuploidy paradox and suggests that aneuploid cells must develop aneuploidy-tolerating mechanisms to cope with CIN and adopt a malignant fate [25]. When sustained, CIN will yield a progeny of cells with variable aneuploid karyotypes that drive tumour evolution and that can help cells to adapt to the initial growth defects imposed by aneuploidy and challenges of the tumour microenvironment. While further work is still required to better understand the interaction between aneuploid cells and the tumour microenvironment [17–20], karyotype evolution provides an important explanation for why CIN is associated with tumour progression, tumour relapse, metastasis and poor prognosis [21–24].

In this review, we aim to give an overview of the initial stresses that aneuploidy imposes on cells, some of the mechanisms that can lead to aneuploidy tolerance and how these mechanisms can potentially be exploited in aneuploid cancer therapy.

## 2. The paradox of aneuploidy in tumorigenesis

Several recent studies have demonstrated a clear relation between aneuploidy and tumorigenesis. For instance, specific trisomies induced in mouse embryonic stem (ES) cells were shown to yield increased neoplastic potential [26], and a study in immortalized mouse embryonic fibroblasts (MEFs) demonstrated that single chromosome losses in tetraploid MEFs led to increased CIN, DNA damage and tumour formation when these cells were transplanted into immunocompromised mice [27]. Aneuploidy is also correlated with enhanced adaptability and malignant transformation of human cells. For example, while aneuploidy suppresses proliferation of human aneuploid DLD1 cells under standard culture conditions, aneuploid DLD1 cells outcompeted their euploid counterparts when placed under less favourable conditions, such as serum depletion, or when cultured in the presence of genotoxic compounds [20]. Similarly, aneuploid clones within human colorectal cancer cultures show a selective advantage and an increase in tumorigenic behaviour under stress conditions [20].

It has also been suggested that aneuploidy in a triploid or tetraploid cell can lead to further chromosomal instability, thereby promoting tumour evolution and tumorigenesis [30,31]. This process might well directly start after tetraploidization as the molecular machinery of tetraploid cells already displays molecular signatures that prepare cells for CIN tolerance. Further work is required to unveil the molecular adaptions that tetraploid cells and their aneuploid descendants undergo on their way to become a cancer cell [32]. Indeed, several studies have shown that the induction of CIN can lead to the development of cancer. For instance, the loss of the mitotic checkpoint components *Mad1* and *Mad2* predisposes mice to chromosomal instability and the development of spontaneous tumours, although tumours are sporadic and occur with long latencies [6,7]. Similarly, the overexpression of the kinetochore protein Hec1 leads to hyperactivation of the mitotic checkpoint, thereby causing CIN and tumorigenesis in mice [33]. Accordingly, mosaic variegated aneuploidy syndrome (MVA) patients, who have mutations in the centrosomic protein *CEP57* or in the SAC protein *BUBR1*, are highly susceptible to childhood cancers, further underscoring that ongoing CIN predisposes to cancer [34,35]. Moreover, CIN is associated with drug resistance, most likely by expediting the generation of new karyotypes that promote tumour cell evolution [36]. For example, it has been shown that colorectal cancers cells that exhibit a CIN phenotype display intrinsic multidrug resistance compared to chromosome stable cell lines [37]. Also in multiple myeloma, CIN has been shown to drive tumour heterogeneity and to underlie acquired drug resistance [38,39]. Finally, CIN can promote tumour relapse. For instance, the induction of CIN by overexpression of *Mad2* greatly promotes tumour recurrence in an inducible K-Ras-driven lung cancer model even when the initial mutant K-Ras driver is alleviated [40].

On the other hand, aneuploidy has been shown to suppress cell growth. For example, the experimental introduction of extra chromosomes in yeast cells revealed that aneuploid cells grow slower than their euploid counterparts due to defects in cell cycle progression, altered metabolic pathways and protein folding distress [14]. Induced aneuploidy in MEFs [15,16] and human cells [41] was also shown to negatively impact proliferation and metabolism and to induce stress responses. Similarly, tumour-suppressive effects have been observed in a CIN setting. For example, reduced expression of the centromere protein *CENP-E* leads to CIN and aneuploidy *in vitro* and *in vivo* and while this mildly predisposes mice to haematopoietic and lung malignancies, CENP-E heterozygous mice are more resistant to chemically and genetically induced tumours suggesting that CIN can also act tumour suppressive [13]. The latter is possibly explained by the fact that the chemical insults and genetic predisposition tested (p19^ARF^ loss) further increase the CIN rate, thus elevating CIN to levels too toxic for cells [42]. Similarly, the reduction of *in vivo BubR1* protein levels enhances the risk for colon cancer but decreases the chance of tumours in the small intestine [43]. Thus, CIN can promote tumorigenesis and restrain tumours, which might depend on the (epi)-genetic context such as the cell type in which the CIN occurs in, but also on CIN rates. For instance, CIN will lead to a reshuffling of oncogenes and tumour suppressor genes and can thereby contribute to cancer genome evolution. However, when provoked in mouse models, CIN has shown variable potency to cause tumorigenesis [44], which might stem from the difference in CIN rates in these mouse models [42], the gene mutations driving the CIN phenotype and the tumour types they develop. Indeed, recent findings are suggesting that CIN rates hold prognostic value for the clinic for several cancers. For example, patients that suffer from ER-negative breast cancer with extremely high CIN rates have a better prognosis than patients with intermediate CIN rates [23]. A similar relationship was found for ovarian cancer, non-small-cell lung cancer and gastric cancer [23]. Accordingly, cancers displaying intermediate copy number variations are associated with the worst overall survival in a pan-cancer analysis [36]. These findings imply that drug-imposed increase of CIN rates could offer a powerful means to treat aneuploid tumours with intermediate CIN rates, increasing CIN beyond the critical point of ‘tolerable’ genomic instability.

However, measuring CIN rates in primary tumours is not trivial [45]. Single-cell whole-genome sequencing (scWGS) is an increasingly popular method to determine intratumour karyotype heterogeneity as an estimate for the CIN rates in a tumour [45]. As such, scWGS hast become a powerful tool to estimate CIN rates in primary tumours aiding to better understand the correlation between CIN levels and clinical prognosis [46], which ultimately might improve therapy stratification.

## 3. Mechanisms underlying aneuploidy tolerance

As discussed above, aneuploidy and CIN decrease the cellular fitness of untransformed cells but are also associated with increased proliferative potential of cancer cells. This suggests that cancer cells can adjust to the aneuploid state through specific survival mechanisms, typically referred to as aneuploidy tolerance mechanisms. The existence of such mechanisms is further supported by *in vivo* studies. For instance, the loss of Mad2 is tolerated by epidermal cells in mouse skin, but hair follicle stem cells are eliminated as a result of apoptotic cell death, which suggests that epidermal basal cells have an aneuploidy-tolerating mechanism in place, while hair follicle stem cells do not [47]. Furthermore, aneuploid cells have been found within a variety of somatic cell types while aneuploidy appears to be less common in stem cells, further suggesting that stem cells have dedicated mechanisms such as special checkpoints to circumvent the propagation of aneuploid cells [48].

**Figure 1.**
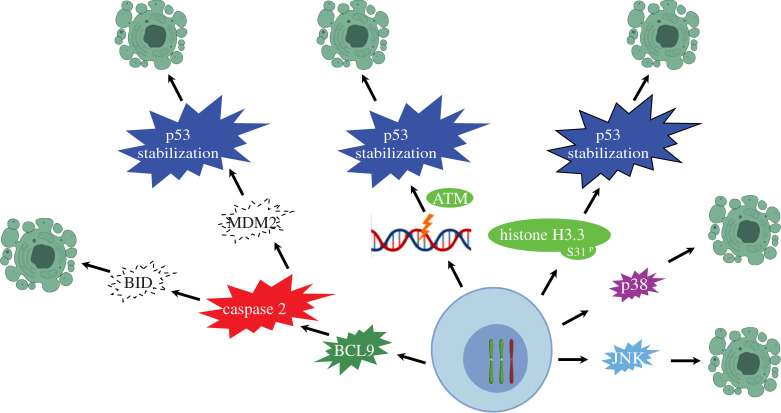
Mechanisms of CIN-imposed cell death. CIN and aneuploidy can trigger apoptosis and therefore, blocking apoptosis serves as an important aneuploidy-tolerating mechanism. Aneuploid cancer cells alter various pathways to overcome CIN-imposed apoptosis, summarized here. Aneuploidy can cause DNA damage, activating DNA damage-induced ATM kinase, following p53-dependent cell cycle arrest and apoptosis. Alternatively, histone H3.3 Ser31 phosphorylation can activate p53 to provoke apoptosis and suppress the proliferation of aneuploid cells. Aneuploidy can also lead to the activation of Caspase-2 when BCL9L is present, which causes cleavage of MDM2 and BID, subsequently leading to p53-dependent and -independent apoptosis. Additionally, aneuploidy can activate p38, resulting in p53-dependent apoptosis, while p38 deficiency upregulates Hif-1*α* to suppress apoptosis. Finally, aneuploidy-induced ROS can induce proliferation or apoptosis through JNK signalling.

One of the most-studied candidate genes to support aneuploidy tolerance is p53 (figure 1). Many studies have found that the p53 pathway favours cells with a diploid karyotype by triggering apoptosis or cell cycle arrest in aneuploid cells [49,50]. Indeed, p53 mutations are frequently observed in highly aneuploid cancers including endometrial, colorectal and gastric cancers. Furthermore, p53-mutant tumours display more complex and unstable karyotypes than p53 wild-type tumours [51,52]. The aneuploidy-suppressive role of p53 is also supported in mouse models for aneuploid cancer. For instance, while CIN alone was found to be a poor instigator of cancer, concomitant p53 inactivation resulted in aggressive and highly aneuploid cancers [53–55]. Likewise, human colon organoids exhibiting a CIN phenotype and harbouring a p53 mutation form more metastases than those without a CIN phenotype [56]. Furthermore, mutations in p53 appear to precede the accumulation of aneuploid cells in Barrett's oesophagus [57]. Finally, p53 has been found to suppress the propagation of structural aneuploidies following chromosome segregation errors [58].

However, what exactly activates p53 following an aneuploidy insult remains controversial. Possible triggers include the DNA damage response, reactive oxygen species and histone H3.3 Ser31 phosphorylation following chromosome missegregation [59–61]. In addition, the stress kinase p38 has recently been found to be required for p53-mediated cell cycle arrest following cytoskeleton disruption [49,62]. Finally, p53 activation could also be a direct consequence of the aneuploid state itself, although it remains elusive how p53 would sense this independently of the aneuploidy-imposed stresses that feed into the p53 signalling pathway.

Overcoming aneuploidy-induced apoptosis is therefore considered an important aneuploidy-tolerating mechanism (figure 1) [50]. For instance, the inhibition of JNK signalling provokes apoptosis of cells displaying a CIN phenotype, presumably due to an impaired DNA damage response [63]. Conversely, in *Drosophila*, JNK activation will trigger apoptosis in cells exhibiting CIN [64]. While in some cases aneuploidy-induced apoptosis is p53 dependent [65], and thus relates to the link between p53 and aneuploidy, in other cases resistance towards apoptosis is acquired through p53-independent mechanisms. For instance, in colorectal cancer, BCL9L dysfunction helps cells cope with aneuploidy by reducing the expression of Caspase-2, thus preventing cleavage of the p53 inhibitor MDM2 and the pro-apoptotic protein BID, effectively blocking the mitochondrial apoptosis pathway [66]. In line with this, Caspase-2^null^ mice are more prone to develop genome unstable lymphoma [67]. Similarly, the oncogenic transcription factor c-Myc can trigger p53-independent apoptosis to remove cells that underwent abnormal mitosis [68]. Finally, yet another, recently uncovered aneuploidy-tolerating mechanism involves MAPK signalling and the p38 stress response kinase. Reduction of p38 activity was shown to upregulate hypoxia-inducible factor HIF-1α, which in turn promotes cell survival following chromosome missegregation by enhancing glycolysis independently of p53 [27]. Altogether these observations suggest that identifying and targeting the aneuploidy-tolerating pathways can be exploited to reduce the fitness of aneuploid cells in tumour development.

## 4. Potential aneuploidy-targeting therapeutic strategies

**Figure 2.**
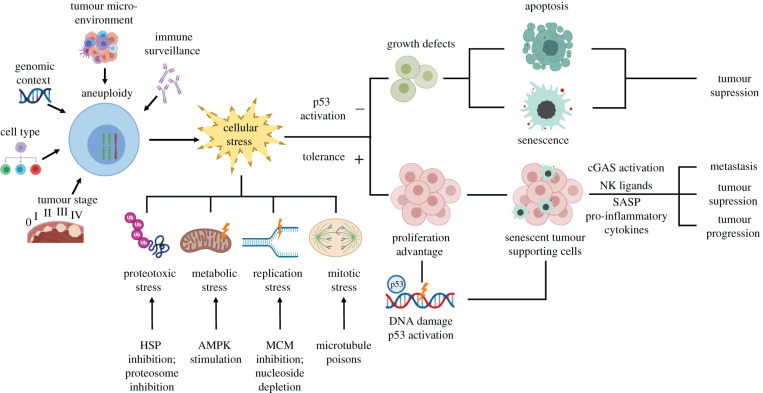
Pathways involved in the consequences of aneuploidy. The role of aneuploidy in tumorigenesis depends on tumour stage, cell type, genomic context, tumour microenvironment and immune response. Aneuploidy promoting detrimental cellular stresses, such as proteotoxic stress (HSP inhibition, proteasome inhibition), metabolic stress (AMPK stimulation), replication stress (MCM inhibition, nucleoside depletion) and mitotic stress (microtubule poisons) can activate apoptosis and senescence, suppressing tumour development. On the other hand, cancer cells can develop tolerance mechanisms to permit the propagation of aneuploid cells. Some proliferating aneuploid cells may stimulate the DNA damage response leading to senescence and cGAS activation. The resulting senescent cells can produce SASPs and the activated cGAS pathway increases pro-inflammatory cytokines to elicit NK cell recognition to suppress tumorigenesis or to promote tumorigenesis or metastasis.

Aneuploidy confers a growth disadvantage to untransformed cells but still is a hallmark of cancer cells. This suggests that cancer cells have adopted mechanisms to cope with the detrimental consequences of aneuploidy, including different responses to cellular stresses, immune system activation and cell cycle arrest as discussed above. As aneuploidy is a hallmark of cancer cells that discriminates healthy cells from cancer cells, such mechanisms make promising targets for cancer therapy, which will be discussed in the context of the consequences of aneuploidy. Figure 2 shows an overview of these stresses and some of the possible interventions.

### 4.1. Enhancing the level of chromosomal instability

Although cells can adapt to aneuploidy through various mechanisms, excessive CIN beyond a critical point will lead to the death of cancer cells. Thus, enhancing the level of CIN has been proposed as a strategy to target aneuploid cancer cells (table 1). Cells with mild levels of CIN were found to be more sensitive to low doses of taxol, which enhanced the number and severity of chromosome segregation errors [69]. Similarly, when CIN rates were increased in glioblastoma-derived tumour initiation cells (TICs) that displayed low intrinsic CIN rates, proliferation was decreased and tumour formation was abolished in an orthotopic mouse model [70]. However, while cancer patients are commonly treated with compounds that increase CIN rates in cultured cells (e.g. vincristine, paclitaxel), the molecular mechanisms driving tumour regression in patients treated with such compounds remain under debate [71]. Several targeted compounds that target mitotic regulators such as *MPS1*, *PLK4* and *AURKA* to exacerbate CIN phenotypes are currently in clinical trials, mostly in phase I [72], some of them with promising first results [73,74]. Other, not yet clinically applied examples include inhibitors of the SAC proteins *Mad2* or *BubR1*, which provoke apoptotic cell death in colorectal cancer cells [75] and the compound INH1, which targets the Hec1/Nek2-related mitotic pathway thus provoking mitotic abnormalities and cell death [76,77]. Likewise, combinations of SAC inhibitors and CIN inducers can synergistically reduce tumour growth. For example, a dysfunctional SAC combined with the microtubule destabilizing drug SKI606 (a Src inhibitor) was found to selectively kill cells with a CIN phenotype [79]. Similarly, the combination of paclitaxel and MPS1 inhibitors was reported to reduce the growth of xenografts *in vivo* much more than either inhibitor alone [78]. Another *in vivo* study showed that combining a p38α inhibitor with taxane-based chemotherapy increased the efficiency of clearing breast cancer cells compared to taxanes alone by boosting chromosome instability [80].

**Table 1.** Evidence for potential aneuploidy-associated actionable vulnerabilities.

		species	cell type	aneuploidization method	karyotype	*in vitro*	*in vivo*	targets	reference
enhancing CIN		human	U2OS, HCT116, LS174-T, HeLa	Mps1 or BubR1 shRNA	random aneuploidy	+	−	low doses of taxol	[69]
		human	HeLa	Tao1 shRNA	random aneuploidy	+	−	TAO1 ↓	[125]
		human	HeLa	Bub1 siRNA	random aneuploidy	+	−	Bub1 ↓	[126]
		human/mouse	patient derived xenografts/MMTV-PyMT tumour model	aneuploid breast cancer cells from patients/Cre-mediated p38α deletion in mouse	random aneuploidy	−	+	p38*α*↓+ taxanes (docetaxel and paclitaxel)	[80]
		human	HeLa, T98G, SW480, DLD-1	Mad2 or BubR1 siRNA	random aneuploidy	+	−	BubR1 ↓ or Mad2 ↓	[75]
		human	MDA-MB-468, SKBR3, T47D, MDA-MB-361, ZR-75-1, MDA-MB-435, HS578T, HBL100, MCF10a	Hec1/Nek1 inhibitor NIH1	random aneuploidy	+	+	Hec1/Nek2 ↓	[76]
		human	HCT-116, HeLa	Mps1 inhibition	random aneuploidy	+	+	MPS1 ↓+ low dose of paclitaxel	[78]
		human	RPE1, MCF7, HT29, SW620	Mad2 shRNA, MPS1 inhibitor reversine/micronuclei mediated chromosome transfer (MMCT)	random aneuploidy/trisomy 5 and 12	+	−	Src ↓+ CIN induction	[79]
		human	Caki-1, U87M	CENP-E siRNA	random aneuploidy	+	+	CENP-E ↓	[65]
targeting cellular stress	proteotoxic stress	human	RPE1	Mad2 shRNA or reversine	random aneuploidy	+	+	UBP3 ↓or USP10 ↓	[127]
		budding yeast		[kar1 × wt] mediated chromosome transfer	disomic strains from [14]	−	+	UBP6	[88]
		human	HCT-116, RPE1	MMCT	trisomies and tetrasomies	+	−	HSP90 ↓or HSF1 ↓	[124]
		*Drosophila*		Mad2 or Rad21 RNAi	random aneuploidy	−	+	Tor ↓	[128]
	metabolic stress	Budding yeast		[kar1 × wt] mediated chromosome transfer	disomic strains from [14]	−	+	dual inhibition of serine and sphingolipid synthesis	[129]
		budding yeast Δ8 strain		acquired aneuploidy	chromosome XI gain	−	+	CCP1̃,UTH1	[95]
		budding yeast		[kar1 × wt] mediated chromosome transfer	disomic strains	−	+	Hxt6/Hxt7	[14]
		*Drosophila*		Mad2 RNAi	random aneuploidy	−	+	PASK ↓	[130], [131]
		*Drosophila*		Mad2 RNAi	random aneuploidy	−	+	PEPCK, G6PD, Cat, Sod1, Idh, Wwox	[131]
		*Drosophila*		Mad2 RNAi	random aneuploidy	−	+	JNK signalling ↓	[63]
		mouse	MEFs	SIRT3 ko + Myc and/or Ras overexpression	acquired aneuploidy	+	+	SIRT3	[132]
		human/mouse	293T, HepG2/MEFs	SIRT4 ko	acquired aneuploidy	+	+	SIRT4	[133]
		human/mouse	DS patient fibroblasts/ DP16 (DS mouse model) MEFs	Down syndrome (DS)	extra copy of human Chr 21 or mouse Chr16	+	+	Nrf2 stabilization	[134]
		mouse	MEFS, T-cell lymphomas	Cre-mediated Mps1 truncation	random aneuploidy	+	+	MPS1↓, p53↓	[54]
	replication stress	human	HCT-116, RKO, HT29, HT55, SW620, SW1116, NCIH747, SKCO1, SW620, T84	CIN^+^ versus CIN^-^ cells	random aneuploidy	+	−	PIGN, MEX3C, ZNF516, Nucleosides ↑	[99]
		human	HCT-116, RPE1	MMCT	tri- and tetrasomies	+	−	MCM2-7	[31]
		human	RPE1	Mps1 siRNA	random aneuploidy	+	−	MPS1	[102]
		human	pluripotent stem cells	acquired aneuploidy during passaging	chromosome 12 or 17 gain	+	−	SRF↑	[135]
	mitotic stress	human	DLD1/amniocytic fibroblasts	MMCT/patient cells	trisomy 7 or 13	+	−	SPG20	[30], [20]
		human	RPE1	Mps1 siRNA	random aneuploidy	+	−	MPS1	[102]
immune system reactivation		human/mouse	U2OS/MEFs	X-ray irradiation	random aneuploidy	+	−	cGAS-STING pathway	[106]
		human/mouse	MCF10A, UWB1.289/B16-F10	X-ray irradiation, genotoxic agents	random aneuploidy	+	+	Genotoxic agents + immune checkpoint blockade	[136]
		human/mouse	MDA-MB-231, H2030/4T1	dnMCAK overexpression	random aneuploidy	+	+	NF-κB pathway	[103]
		human/mouse	Tumour and matched peritumoral specimen from gastric cancer patients, BGC-823, SGC-7901/chronic *H. pylori* infection mouse model	n.a.	n.a.	+	+	STING ↑	[111]
senescence induction		human	RPE1, HCT116, U2OS	Nocodazole, reversine, Bub1 or Smc1a shRNA	random aneuploidy	+	+	MPS1, BUB1 and SMC1A	[114]
		human	MKN45, ST2957	Mad2 or BubR1 shRNA	random aneuploidy	+	−	Mad2 ↓or BubR1 ↓ + PTX	[122]
		human	IMR90, MCF10A	Mad2 siRNA	random aneuploidy	+	−	Mad2	[116]

While these observations clearly show that enhancing CIN could be a powerful method to eradicate CIN tumours, the feasibility of such therapies depends on many factors including CIN status and CIN tolerance, many of which need further study before we can be certain that enhancing CIN is a fully safe approach to target CIN cancers.

### 4.2. Targeting the cellular stresses imposed by aneuploidy

Instead of targeting the process of chromosome missegregation itself, specific vulnerabilities caused by aneuploidy-associated cellular stresses such as proteotoxic, metabolic, replication and mitotic stress can potentially be exploited in therapy as well, such as by directly reverting this adaptation or by enhancing these stresses beyond tolerable levels (table 1), which we will discuss further below [65,81,82].

Aneuploid cells display proteotoxic stress, which includes increased protein degradation [83] and aggregation [84], as well as impaired protein folding [85]. This aneuploidy-imposed stress is caused by changes in protein levels that are produced by genes on the aneuploid chromosomes [86] and which lead to imbalances in the protein complex stoichiometry [41,87]. Indeed, reducing proteotoxic stress improves the survival of aneuploid cells. For instance, the loss of the deubiquitinating enzyme (DUB) UBP6 improves survival of aneuploid yeast strains by increasing proteasome-mediated protein degradation and thus reducing proteotoxic stress [88]. This poses a targetable vulnerability, as some aneuploid yeast strains show increased sensitivity to the proteasome inhibitor MG132 and the loss of the deubiquitinase Ubp3, a DUB that is required for full proteasome function [11,76].

In addition to the increased protein burden itself, induced aneuploidy also impairs HSP90-mediated protein folding, further increasing proteotoxic stress. Increasing protein levels of heat shock factor 1 (HSF1) counteract this effect, revealing HSF1 overexpression as an aneuploidy-tolerating hit in human cells [85]. These observations can possibly be exploited in therapy, such as by treating aneuploid cancers with an HSP90 protein folding inhibitor (17-AAG, 17-allylamino-17-demethoxy-geldanamycin) or drugs inhibiting HSF1 activation [83], thus effectively boosting proteotoxic stress in aneuploid cells beyond tolerable levels [89]. Indeed, the HSP90 inhibitor 17-AAG has significant anti-tumour activity [90], and when combined with the energy stress-inducing compound AICAR, 17-AAG is particularly toxic to aneuploid cells [91]. Thus, while eliminating proteotoxic stress is beneficial for aneuploid cells, (pharmaceutical) exacerbation of proteotoxicity might be a promising new avenue for cancer therapy.

Autophagy, a process involved in the removal of damaged or surplus proteins and organelles is upregulated in aneuploid cells. For instance, human colon cancer cells carrying an extra chromosome display increased LC-3 foci, a marker for autophagy, compared to control cells [41]. In concordance, aneuploid cells are more sensitive to autophagy inhibitors [92] such as chloroquine, a compound that inhibits late stages of autophagy. Chloroquine was shown to preferentially inhibit proliferation of trisomic MEFs compared to euploid MEFs [93]. Similarly, trisomic MEFs showed impaired proliferation when another autophagy factor, Beclin 1, was knocked down [91]. Furthermore, aneuploid cells show increased expression of the cytosolic receptor SQSTM1, a protein that targets ubiquitinated proteins to the autophagy machinery further exemplifying how aneuploid cells depend on autophagy [94]. Altogether, these findings indicate that interfering with autophagy could be another promising route towards selective aneuploidy-targeting therapy [83].

In addition to proteotoxic stress, aneuploid cells also suffer from metabolic stress *in vitro* as well as *in vivo* [14,15,41,54,91], which provides another targetable vulnerability. For instance, as mentioned above, trisomic MEFs are much more sensitive to the energy stress-inducing compound AICAR than their euploid counterparts [92]. Furthermore, increased proliferation of aneuploid cells coincides with an increase in the levels of sphingolipids, and conversely, dual inhibition of serine and sphingolipid synthesis is lethal to aneuploid yeast cells [95]. The upregulated metabolism observed in aneuploid cells frequently coincides with increased levels of reactive oxygen species (ROS), which can activate the DNA damage response [96]. In non-transformed cells, this can be toxic, as shown in *Drosophila*, in which aneuploidy-induced ROS triggers JNK activation and subsequent apoptosis [97]. Together, these findings suggest that exacerbating the metabolic phenotype of aneuploid cells could be yet another way to selectively kill aneuploid cancer cells.

Aneuploidy has also been associated with the downregulation of replication factors, in particular the subunits of the replicative helicase MCM2-7 [31,98]. The resulting replication stress can lead to extra-chromosomal instability, such as an increase in the frequency of anaphase bridges as observed in aneuploid HCT116 and RPE1 cells. Restoring the expression of MCM2-7 back to wild-type levels partially rescues this phenotype [31]. Similarly, colorectal cancer cells exhibiting a CIN phenotype also suffer from high levels of replication stress, which leads to further DNA damage [99]. Supplementing these cells with nucleosides reduces both DNA damage and segregation errors [99]. Together, these observations indicate that replication stress as a result of aneuploidy will further increase the CIN phenotype and thus tumour heterogeneity. While inhibiting aneuploidy-imposed replication stress might have limited effects on established cancer cells, enhancing replication stress could push aneuploid cells over the edge, which could be another means to selectively kill CIN cells [99,100].

Lastly, the gains and losses of chromosomes will also affect the expression of mitotic proteins including the machinery of the SAC and chromosomal segregation. The resulting mitotic stress might be a reason that stable aneuploidy leads to CIN and thereby the generation of new karyotypes. For instance, lymphocytes from individuals born with systemic and stable trisomies for either chromosome 13, 18 and 21 show an increased frequency of aneuploidies for three other autosomes (chromosomes 8, 15 and 16) compared to lymphocytes of healthy controls suggesting that stable aneuploid cells tend to destabilize their genomes [101]. Similarly, DLD1 colorectal cancer cells carrying an extra chromosome 7 or 13 display reduced mitotic fidelity compared to diploid DLD1 cells [30], further suggesting that aneuploidy can induce chromosome missegregation.

Taken together, these findings indicate that aneuploidy-imposed protein imbalances lead to (i) proteotoxicity (protein misfolding, protein aggregation and proteasomal degradation), (ii) increased autophagy, (iii) an increased cellular metabolism, (iv) increased replication stress and (v) a deregulated mitotic machinery, which all offer promising therapeutic targets. However, further research into the effects of deregulating these pathways in aneuploid cells is still needed, particularly as exacerbating the toxic effects of CIN might also lead to malignant transformation of non-aneuploid cells. Vice versa, aneuploidy-targeting therapies could also lead to the selection of near-euploid cancer cells or aneuploid cancer cells that no longer rely on the targeted pathway to cope with CIN, both of which would lead to cancer recurrence. However, further work, including extensive single-cell DNA and RNA sequencing, is required to better understand which evolutionary paths CIN tumours exploit to become therapy resistant.

### 4.3. Activating the immune system to target aneuploidy

In addition to targeting cell-intrinsic consequences of aneuploidy, the tumour microenvironment can potentially also be exploited to clear aneuploid cancer cells. More specifically, (re)activation of the immune system can become a new therapeutic strategy to treat aneuploid tumours since aneuploid cells might be recognized by the innate immune system (table 1) [102]. Aneuploid cells with complex karyotypes trigger an upregulation of pro-inflammatory factors [32,102] and are cleared by natural killer cells in a co-culture setting [102]. In order to survive and transform into an aneuploid cancer cell, aneuploid cells need to circumvent this clearance mechanism, but how aneuploid cancer manage to do this *in vivo* is not yet clear. One candidate mechanism to be involved is cGAS-STING signalling. Missegregated chromosomes often localize into micronuclei which, when ruptured, release genomic DNA into the cytosol. This leads to the activation of cyclic GMP-AMP synthase (cGAS), a major cytosolic nucleic acid sensor with dsDNA as its ligand [103–106]. cGAS activation generates cyclic dinucleotide cyclic GMP-AMP (cGAMP), which in turn activates a Type I Interferon response and initiates NF-κB signalling via the adaptor Stimulator of Interferon Genes (STING) [107,108]. This axis thus acts tumour suppressive and, indeed, several cancers display decreased cGAS-STING signalling, including colorectal carcinoma and melanoma [109,110], which has been associated with poor survival [111]. Cancer cells may thus have found a way to circumvent activation of the immune system upon micronuclei rupture. On the other hand, cGAS was also found to inhibit homologous recombination-mediated DNA repair, thereby decreasing the efficiency of DNA repair and thus promoting tumorigenesis [112]. Furthermore, active cGAS-STING signalling promotes metastasis of human triple-negative breast cancer cells in athymic mice [103]. The cGAS-STING pathway thus seems to have a tumour suppressive as well as a tumour-promoting role. Further work is required to resolve this apparent paradox and should determine in which setting cGAS-STING inhibition or activation is the best strategy to kill aneuploid cancer cells [113].

### 4.4. Targeting aneuploidy by induction of senescence

Senescence is a state of irreversible growth arrest without cell death. Senescence can be induced by unrepaired DNA damage or other cellular stresses that yield a robust p53 response [114]. As many cancer therapies provoke DNA damage, therapy-induced senescence has been proposed as a promising strategy to treat cancer (table 1), particularly when combined with senolytic drugs (i.e. drugs that next selectively eliminate the therapy-induced senescent cells [115]).

Numerous studies over the past decades have found that cellular senescence is a frequent event in CIN cell populations. For instance, deletion of the SAC genes *BUB1* and *MAD2* can trigger a senescence phenotype [116,117]. Similarly inactivation of SMC1A, a component of the Cohesin complex, leads to aneuploidy and senescence [118]. Aneuploid cell populations secrete cytokines that have been associated with the senescence-associated secretory phenotype (SASP) [119] and can trigger an immune response [102]. Therefore, exacerbating aneuploidy in cells with a CIN phenotype in combination with senolytics could become a new strategy to eradicate aneuploid cancer cells *in vivo*.

Senescence is generally considered as a barrier to malignant transformation; however, the cytokines secreted by senescent cells have two opposing roles in the tumorigenesis. On the one hand, they will trigger cytokine release of the surrounding cancer cells and immune-mediated clearance thus suppressing tumour development [102]. On the other hand, the secreted cytokines can also accelerate age-associated phenotypes and metastasis [120,121]. Both scenarios appear to occur in human cancer. For instance, a recent study showed that provoking complex karyotypes (i.e. more than five chromosomes gained or lost per cell) in several cell lines resulted in senescence and SASP, which led to the increased invasion, migration of the cancer cells *in vitro* and *in vivo* and angiogenesis *in vivo* [114]. Conversely, another study found that when a senescent state was induced in gastric cancer cell lines by silencing Mad2 and BubR1, this led to decreased cell proliferation, migration and invasion and this phenotype was aggravated when the CIN rates were further increased by concomitant treatment with the microtubule poison paclitaxel [122]. In line with the latter, the elimination of chemotherapy-induced senescent cells was found to reduce the risk of cancer recurrence and furthermore associated with reduced chemotherapy-associated bone marrow suppression and cardiac dysfunction [123]. Taken together, it is still not fully understood when senescent cancer cells should be eliminated and when not. This probably relies on the specific context of the SASP or cancer type.

## 5. Concluding remarks

In this review, we discussed the paradoxical role of the consequences of aneuploidy as tumour-promoting or tumour-suppressing features. Although in most cases aneuploidy is detrimental for cells, it confers a fitness advantage under some circumstances. This probably depends on the (epi)-genetic context such as the cell type in which the aneuploidy occurs, but also on the CIN rates within the cells. Thus, before targeting aneuploid cancers by increasing genomic instability, both the context as well as the pre-existing CIN rates should be carefully considered. One way to estimate *in vivo* CIN rates is by determining the level of intratumour karyotype heterogeneity, for instance by single-cell whole-genome sequencing [45]. When considering increasing CIN rates as a therapeutic approach, it is furthermore important to consider that untransformed cells will also be affected by the CIN-provoking agents and thus will suffer from low to moderate CIN rates as well, thereby predisposing these cells to become tumorigenic and lead to therapy-induced cancers later on. Therefore, future work should look into how to more selectively target cells displaying a CIN phenotype, either by drug-mediated CIN exacerbation or by (co-)treatment with drugs that exploit other vulnerabilities of CIN cells.

Several of the aneuploidy stress responses have been discussed in this review. However, there is mounting evidence that aneuploid cancer cells have activated tolerance mechanisms to adapt to the detrimental outcome of these stress responses. For instance, the discovery that aneuploid cells have downregulated HSP90 mRNA and protein and are impaired in HSP90-mediated protein folding led to the finding that aneuploid cells are very sensitive to HSP90 inhibitors [124]. It is therefore of the utmost importance to better understand how cells adapt to and cope with an aneuploid DNA content as this will probably reveal more approaches to selectively target cells with a CIN phenotype. In addition to studying the pathways that help cells cope with an aneuploid state, we should also look further into which mutations in aneuploid cancers help aneuploid cells to convert into aneuploid cancer cells as these are also promising targets for aneuploid cancer therapy. Similarly, (re)activation of the immune system could become a new therapeutic strategy to treat aneuploid tumours since aneuploid cells might be recognized by the innate immune system and aneuploid cancer cells might have blocked this response. Future work should reveal whether this response also takes place *in vivo* and how a failing immune response could be reinstated. Lastly, we need to further unravel the relationship between aneuploidy and senescence. Indeed, inducing senescence by DNA damage or provoking CIN has been suggested to be an effective way to treat cancer. However, the role of senescent cells in cancer is still controversial: in some contexts, it is tumour inhibiting, in others tumour promoting. Therefore, future studies should investigate whether senescent cells should be eliminated or not, which probably depends on the individual cancer type.

## Data accessibility

This article has no additional data.
